# Accuracy in identifying the elbow rotation axis on simulated fluoroscopic images using a new anatomical landmark

**DOI:** 10.1007/s11751-017-0289-3

**Published:** 2017-06-07

**Authors:** J. K. Wiggers, R. M. Snijders, J. G. G. Dobbe, G. J. Streekstra, D. den Hartog, N. W. L. Schep

**Affiliations:** 1 Trauma Unit, Department of Surgery, Academic Medical Center, Meibergdreef 9, 1105 AZ Amsterdam, The Netherlands; 2 Department of Biomedical Engineering and Physics, Academic Medical Center, Amsterdam, The Netherlands; 3 Trauma Research Unit, Department of Surgery, Erasmus Medical Center, Rotterdam, The Netherlands; 4 Department of Surgery, Maasstad Hospital, Rotterdam, The Netherlands

**Keywords:** Fluoroscopy, Elbow, Rotation axis, Landmark, Segmentation

## Abstract

External fixation of the elbow requires identification of the elbow rotation axis, but the accuracy of traditional landmarks (capitellum and trochlea) on fluoroscopy is limited. The relative distance (RD) of the humerus may be helpful as additional landmark. The first aim of this study was to determine the optimal RD that corresponds to an on-axis lateral image of the elbow. The second aim was to assess whether the use of the optimal RD improves the surgical accuracy to identify the elbow rotation axis on fluoroscopy. CT scans of elbows from five volunteers were used to simulate fluoroscopy; the actual rotation axis was calculated with CT-based flexion–extension analysis. First, three observers measured the optimal RD on simulated fluoroscopy. The RD is defined as the distance between the dorsal part of the humerus and the projection of the posteromedial cortex of the distal humerus, divided by the anteroposterior diameter of the humerus. Second, eight trauma surgeons assessed the elbow rotation axis on simulated fluoroscopy. In a preteaching session, surgeons used traditional landmarks. The surgeons were then instructed how to use the optimal RD as additional landmark in a postteaching session. The deviation from the actual rotation axis was expressed as rotational and translational error (±SD). Measurement of the RD was robust and easily reproducible; the optimal RD was 45%. The surgeons identified the elbow rotation axis with a mean rotational error decreasing from 7.6° ± 3.4° to 6.7° ± 3.3° after teaching how to use the RD. The mean translational error decreased from 4.2 ± 2.0 to 3.7 ± 2.0 mm after teaching. The humeral RD as additional landmark yielded small but relevant improvements. Although fluoroscopy-based external fixator alignment to the elbow remains prone to error, it is recommended to use the RD as additional landmark.

## Introduction

Hinged external elbow fixation is used to treat persistent instability of the ulnohumeral joint, either following closed reduction of an elbow dislocation or following operative treatment of complex elbow fractures. This treatment theoretically mitigates postoperative stiffness because it allows immediate active and passive motion of the elbow joint, while the joint remains stable [1–6].

Though encouraging outcomes have been reported with external fixators, complications are numerous, including nerve injury, deep infection, increased motion resistance, pin site infection, pin loosening and pin breakage [7]. Some of these complications are explained by incongruence between the rotation axis of the fixator hinge and the anatomical rotation axis of the elbow [7–9]. There are two explanations for this incongruence. First, the elbow rotation axis has an ‘instant center of rotation,’ meaning that the rotation axis is not fixed in three-dimensional space but moves like a twist around a screw. Therefore, it is impossible to place a hinged fixator in perfect alignment with the rotation axis of the elbow, as the latter migrates during flexion and extension. The second reason for incongruence is that surgeons often misidentify the correct elbow rotation axis during surgery, which is potentially preventable.

To position the axis of the fixator hinge, it is essential to identify the elbow rotation axis on fluoroscopy and drill an axis pin (Kirschner wire) through it. However, we showed in a previous fluoroscopic simulation study that the intraoperative accuracy to identify the elbow rotation axis is low and associated with substantial error [10]. Madey et al. [10] showed that applying an external fixator with 5° or 10° incongruence relative to the elbow axis, which was a common error in our previous fluoroscopic simulation study, results in a 3.7- and 7.1-fold increase in motion resistance, respectively [8]. Such incongruence often results in morbidity and secondary procedures. In a recent prospective study of hinged external elbow fixation, 19% of patients had elbow incongruence resulting from fixator malalignment, and these patients all required secondary procedures for fixator realignment or replacement [6].

To identify the elbow rotation axis on fluoroscopy, it is required to obtain an ‘optimal lateral image,’ which should be orientated perpendicular to the rotation axis (i.e., an on-axis image). Traditionally, surgeons aim to overlap the capitellum and the trochlear sulcus until these structures form concentric circles with the centers of these circles representing the axis of rotation [9]. However, orientation with this method alone is limited to the coronal plane (abduction/adduction) and arguably causes rotational errors in the transverse plane (internal/external rotation). In other words, the circles of the capitellum and trochlea can overlap, while there is still unwitnessed rotational error of the lateral image in the transverse plane, as previously demonstrated in a study by Bottlang et al. [11].

**Fig. 1:**
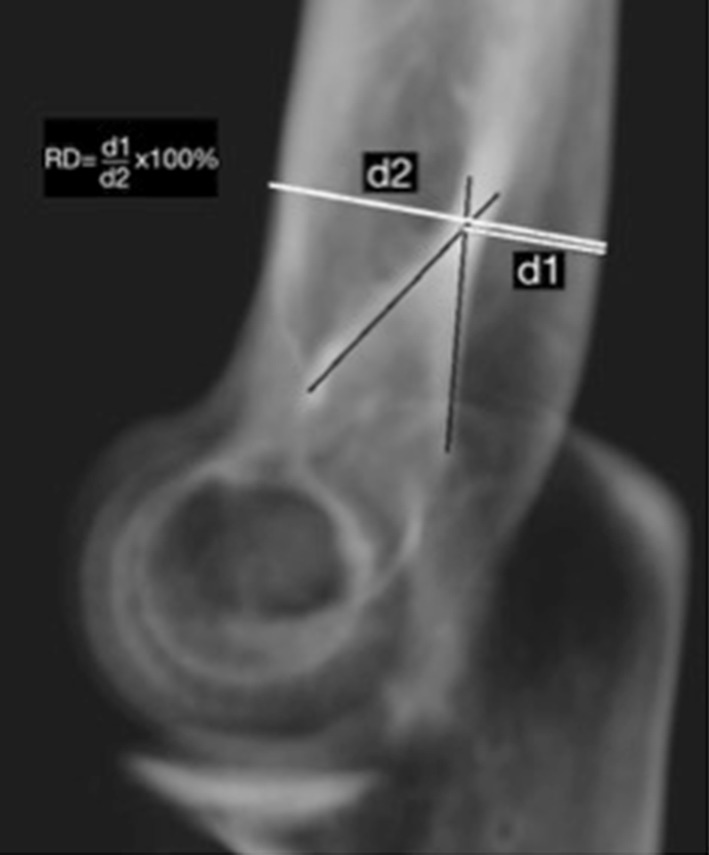
Digitally reconstructed radiograph (DRR) of the humerus in lateral view, depicting how the relative distance (RD) of the humerus is measured. The RD is defined as RD = (*d*1/*d*2) × 100%, with *d1* the distance from the dorsal side of the humerus to the projection of the posteromedial cortex (measured at the intersection point of the cortical lines, as represented by the intersection of the drawn black lines in the figure) (mm), and with *d2*, anteroposterior diameter of the humerus (mm). The *lines d1* and *d2* are measured perpendicular to the bone axis. Finally, the RD is calculated as the length ratio of *d1* and *d2* and expressed as a percentage

Additional radiographic landmarks may improve identification of the optimal lateral image and elbow rotation axis and may eventually improve external fixator alignment. A landmark that could help orientation in the transverse plane is the relative distance (RD) of the humerus [11]. This landmark, developed by Bottlang et al., is based on the relative position of the dense projection of the posteromedial cortex within the boundaries of the distal humerus. The RD is obtained by measuring the distance between the dorsal side of the humerus and the projection of the posteromedial cortex, subsequently dividing this distance by the diameter of the humerus (Fig. 1). Bottlang et al. [11] designed this measure in a study with cadaveric bones and electromagnetic motion tracking data, but these measures have not been validated in healthy volunteers.

In the first part of this study, we determined the elbow rotation axis using 3D image analysis in five healthy volunteers and subsequently assessed the RD value that corresponds to the optimal lateral fluoroscopic image of the humerus in vivo. The second part of this study was designed to assess potential improvements in surgical accuracy to identify the elbow rotation axis, after surgeons have been instructed how to use the optimal RD value as additional landmark on fluoroscopy.

## Methods

### Compliance with ethical standards

This study was approved by the local ethical committee, and was conducted in accordance with the Declaration of Helsinki. Informed consent was obtained from all individual participants included in the study.

### Data acquisition

The non-dominant left elbow of five healthy male volunteers with normal elbow function and no history of trauma, was CT-scanned in incremental flexion angles (0°, 35°, 65°, 100°, 135°) [12]. Scans were made using a Brilliance 64-channel CT scanner (Philips Healthcare, Best, the Netherlands) (120 kV, slice thickness 0.9 mm, increment 0.33 mm, isotropic voxel spacing of 0.33 mm). In the neutral position (0°), the elbow was scanned at a high dose (150 mAs) for adequate virtual modeling of the bone by image segmentation, and at a low dose (50 mAs) for the remaining states of elbow flexion, to limit the radiation expose.

### Calculation of the actual elbow rotation axis

The actual elbow rotation axis is calculated from the CT scans with the elbow in different states of flexion, as described previously [10]. In short, the humerus and ulna were manually segmented from a high-dose scan at 0° flexion, and subsequently aligned, by 3D image registration, with low-dose CT images containing the elbow in subsequent states of flexion. Taking the humerus as fixed reference bone, the ulna will now show a rotation between the segmented state, at 0° flexion, and its position after registration to each of the subsequent flexion images. These rotations evolve about their respective so-called helical axes. Since the helical axes found for elbow rotation between 0° and incremental flexion do not completely overlap due to the previously mentioned ‘instant center of rotation’ of the elbow, we used the average of the four helical axes as the elbow rotation axis, referred to as the ‘calculated rotation axis’ in this study.

### Measuring the in vivo relative distance

**Fig. 2:**
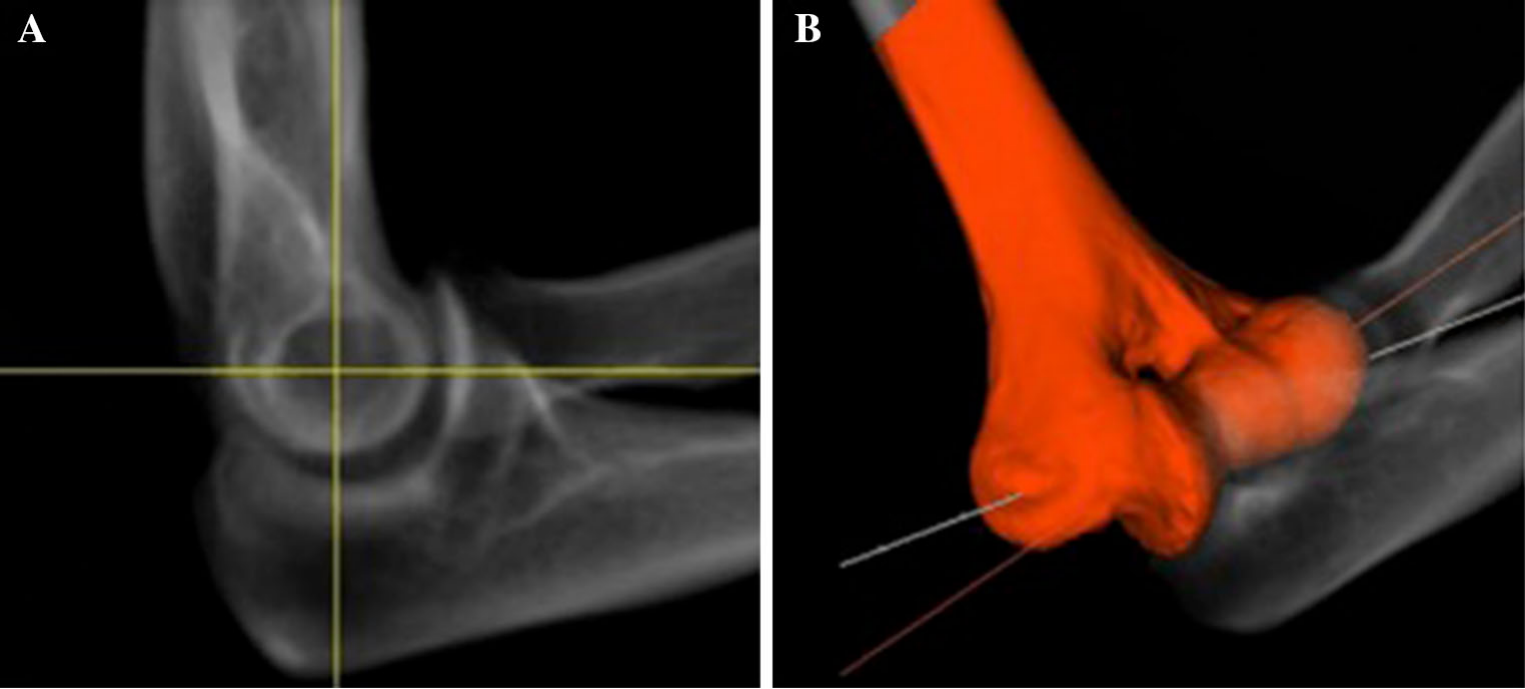
**a** Digitally reconstructed radiograph (DRR) that simulates fluoroscopic images. The figure shows an optimal lateral image of the elbow that is orientated perpendicular to the rotation axis. Surgeons were able to freely rotate the elbow CT to generate DRRs from different projection angles in search of this optimal lateral image and used the crosshair cursor to indicate the position of the rotation axis, **b** example of an axis estimated by one of the surgeons (*red line*) and the calculated rotation axis (*white line*) in a 3D reconstructed image, showing the surgeons’ error

In the first part of this study, we measured the RD that corresponds to an optimal lateral fluoroscopic image of the humerus in vivo. Digitally reconstructed radiographs (DRRs) of the CT scans were used to simulate fluoroscopic images (Fig. 2). Each DRR could be projected into the plane perpendicular to the calculated rotation axis, hence providing an optimal lateral image of the elbow. Because the actual elbow rotation axis may slightly vary over the flexion–extension trajectory, we constructed two evaluation sets of optimal lateral elbow images, both based on the same average elbow rotation axis: one set including images of the five elbows in extension (0° flexion) and one set including images of the five elbows in 100° flexion. Subsequently, three observers (RS, JD and GJ) measured the RD in the two sets of elbow images. Figure 1 shows how the RD was measured.

### Accuracy of assessing the rotation axis using the relative distance

In the second part of this study, we assessed potential improvements of surgeons in finding the elbow rotation axis on fluoroscopic images after the surgeons have been instructed to use the RD of the humerus in addition to traditional landmarks. A custom-made software application was used to simulate fluoroscopy of the elbow. The application produces real-time DRR images and enables the operator to freely rotate and translate the CT volume containing the elbow [13]. Operators are enabled to position the CT volume until the resulting elbow DRR is felt to represent the optimal lateral image perpendicular to the elbow rotation axis. Operators then center the image at the expected position of the rotation axis, and an ‘axis definition’ is subsequently ejected at the crosshair cursor position (Fig. 2).

**Fig. 3:**
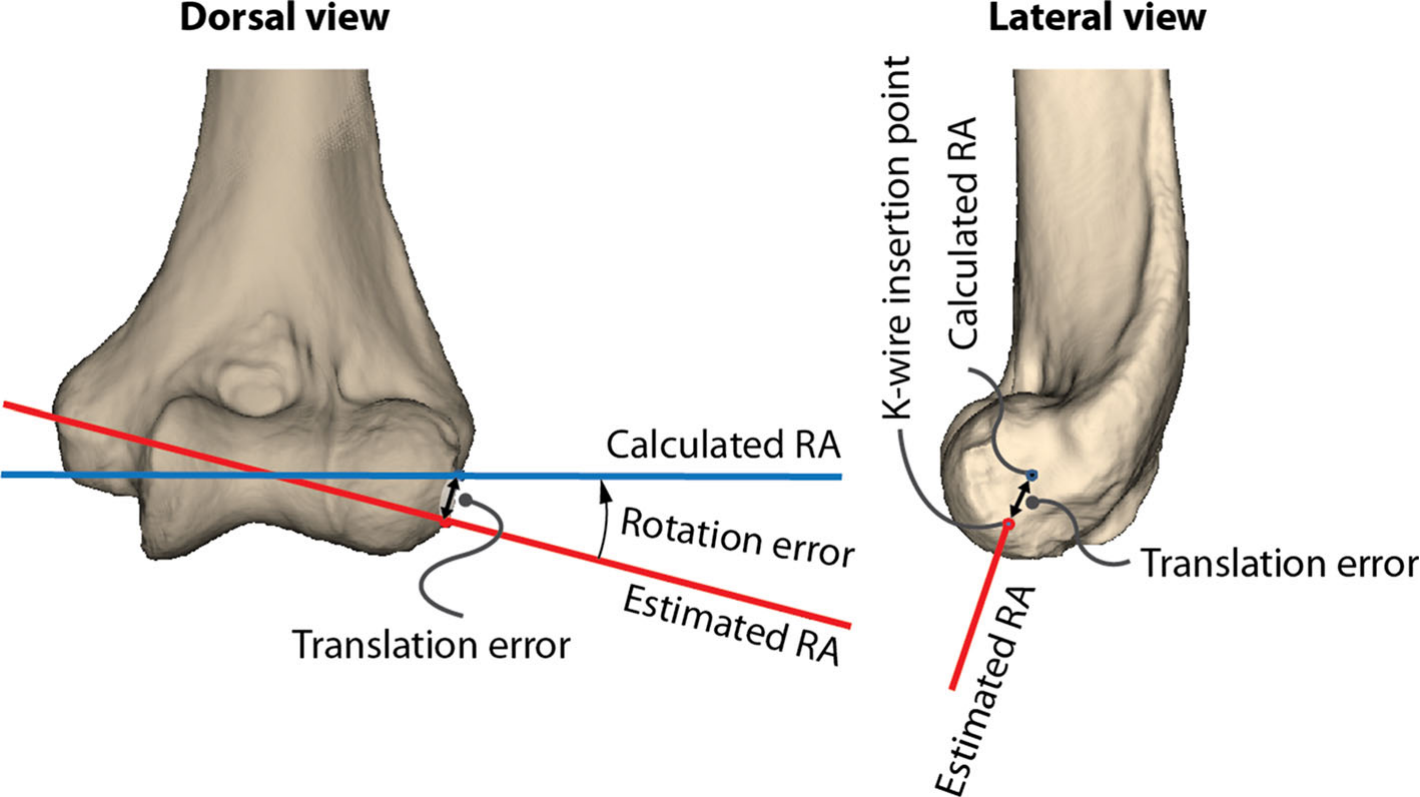
Dorsal and lateral view of the humerus showing the calculated elbow rotation axis (*blue*) and the rotation axis estimated by the surgeon (*red*) by inserting a K-wire. The deviation from the calculated axis is expressed in terms of a rotation error and a translation error. The rotation error describes the projection angle between both axes, while the translation error is defined by the Euler (shortest) distance between the K-wire insertion point and the entry point that corresponds to the calculated rotation axis on the lateral epicondyle

Eight surgeons were invited to determine the elbow rotation axis on simulated fluoroscopy images in two sessions. During each session, the five available scanned elbows were presented three times in random order and each time with a different starting image, resulting in 15 axis definitions per surgeon for each session. In the first session, surgeons were instructed to use traditional landmarks that they normally use in clinical practice, including the overlapping centers of the capitellum, trochlear sulcus and trochlea. After completion of the first session, the surgeons were instructed how to use the humeral RD as anatomical landmark, including the RD corresponding to an optimal lateral image as defined in the first part of the study. Teaching consisted of a 10-min lecture with explanatory figures how to use the humeral RD as landmark. The figures used during teaching were similar to Figs. 1 and 2. After teaching and a break of approximately 30 min, the surgeons conducted the second session of the experiment. They again determined the elbow rotation axis on fluoroscopy, now using the humeral RD as a landmark in addition to the traditional landmarks. All axis definitions provided by the surgeons (i.e., from both the first and second session) were compared with the calculated rotation axis, which provided measures for off-axis alignment by the surgeons before and after teaching of the humeral RD. Off-axis alignment was expressed as rotation error, which is a measure of orientation and expressed as an angle, and as surface translation error, which is measured on the surface of the lateral epicondyle and expressed in millimeters (Fig. 3) [10, 11]. The surface translation error is defined by the Euler (shortest) distance between the entry point of the elbow rotation axis on the lateral epicondyle and the location where the surgeon’s axis definition enters the lateral epicondyle, and thus represents the ‘K-wire insertion error’ if a surgeon would normally start drilling the fixator axis at this location. Finally, the mean rotation and translation error values were compared between the first (preteaching) and second (postteaching) experiment session.

### Statistical analysis

The RD corresponding to an on-axis lateral image of the elbow (i.e., ‘the optimal RD’), as measured by three observers, is expressed as an average with corresponding standard deviation (SD). Correlation between the optimal RD for elbows in flexion and extension was analyzed with a Pearson correlation coefficient; interobserver agreement was assessed with an intraclass correlation coefficient.

Paired-sample *t* tests were used to compare the mean error parameters between elbow axis determination with and without the RD as additional assessment parameter (i.e., preteaching and postteaching).

## Results

### Optimal in vivo relative distance

The mean optimal RD measured 45.9% (SD 5.0) for elbows in extension and 45.6% (SD 5.6) for elbows in flexion. A difference of the optimal RD for elbows in extension and flexion could not be detected (*P* = 0.94); measurements of the optimal RD for elbows in extension and flexion had strong correlation (correlation coefficient 0.80) [14]. The intraclass correlation coefficients for measuring the optimal RD of elbows in extension and flexion were 0.82 and 0.90, respectively, which showed strong interobserver agreement [15].

### Improvements in Surgical Accuracy

The first and second experiment sessions both resulted in 120 axis definitions (8 surgeons × 5 specimens × 3 axis definitions). All surgeons’ axis definitions were compared with the CT-based calculated rotation axis, as illustrated in Fig. 2b. The mean rotational error in identifying the elbow rotation axis decreased from 7.6° (SD 3.4; range 0.61–17.66) before teaching to 6.7° (SD 3.3; range 0.37–16.50) after teaching (i.e., after surgeons had been instructed how to use the optimal RD as additional landmark) (*P* = 0.03). The mean translational error decreased from 4.2 mm (SD 2.0; range 0.78–11.46 mm) before teaching to 3.7 mm (SD 2.0; range 0.23–9.33) after teaching (*P* = 0.01).

## Discussion

Hinged external elbow fixation enables early mobilization after complex elbow dislocation and residual instability, but alignment of the fixator may cause complications and require revision procedures [6]. In this study, we assessed the RD of the humerus as an additional landmark to identify the elbow rotation axis on fluoroscopy images. The technique is easy to use intraoperatively and does not require extra equipment. First, we showed that the in vivo RD corresponding to an optimal on-axis lateral fluoroscopic image averaged 45%. We also showed that measurement of the optimal RD was robust, as evidenced by good interobserver agreement and high correlation between measurements for elbows in extension and flexion. Secondly, we showed that a 10-min teaching program with explanatory figures about the use of the optimal RD (Figs. 1, 2) improved the surgical accuracy in determining the elbow rotation axis, albeit these improvements were small.

Bottlang introduced the RD as an anatomical landmark and suggested the RD should read 27% ± 3.7% to find the optimal lateral image in the transverse plane. The difference in optimal RD between that study and the present study can be explained by the fact that Bottlang used cadaveric bones, whereas the present study was based on simulated in vivo elbow fluoroscopy. Moreover, Bottlang used electromagnetic motion tracking data to determine the elbow rotation axis, but the present study incorporated a CT segmentation technique that has proven to be accurate with rotational errors of (mean ± SD) 0.1° ± 0.1° and translation errors of 0.4 ± 0.1 mm [13]. Our technique of scanning elbows with incremental values of flexion was similar to other recent anatomical studies analyzing elbow rotation axis kinematics [12]. Nonetheless, it seems preferable to validate the determined optimal RD in future studies.

This study showed only a marginal improvement of the surgical accuracy in identifying the elbow rotation axis, but these improvements may still be clinically relevant. This is illustrated in a cadaveric electromagnetic tracking study by Madey et al. [8], who found a linear relation between fixator malalignment and motion resistance. Reducing the rotational error of fixator alignment from 10° to 5° resulted in a 50% decrease in elbow motion resistance. Reducing the rotational error toward a perfect alignment further reduces motion resistance.

Surgical errors in elbow axis definitions were expressed as rotation and translation errors. The rotation error measures the angle between the axis chosen by the surgeon and the calculated rotation axis. The surface translational error represents the shortest distance between the entry points of the axis chosen by the surgeon and the calculated rotation axis on the lateral epicondyle. Both measures are informative of surgical achievements, the latter especially because it provides the distance of the ‘K-wire insertion error’ if a surgeon would normally start drilling a K-wire to position the fixator axis at the chosen point at the lateral epicondyle. Our previous study measured translational error at the shortest distance anywhere between the calculated elbow rotation axis and the surgeons’ axis definition [10]. However, that method is less informative and provides an underestimation of the true translation error.

In this study, the surgeons identified the rotation axis in virtual space but did not actually insert a K-wire. In that respect, the reported surgical errors reflect the X-ray projection that they chose and not their K-wire orientation. This may have underestimated the real intraoperative error, since the actual placement of the K-wires while using fluoroscopy intermittently may add to even larger surgical errors. The study is also limited by its simulation design: we used DRR images to simulate fluoroscopy instead of using real intraoperative fluoroscopy. Nonetheless, the DRR images were designed to resemble the quality of intraoperative fluoroscopy, and the study setting allowed surgeons to freely rotate and translate the elbow similar to the intraoperative setting. One other limitation is that surgeons may get better at determining the axis of rotation with each iteration, which may have biased the improvement in accuracy after teaching surgeons how to use the RD. Furthermore, the study was limited by a low sample size of elbow specimens that were tested, so results regarding significance of the data should be interpreted with caution. It was not possible to increase the number of elbow specimens because it was regarded unethical to subject additional volunteers to the radiation of CT scans. Instead, we tried to circumvent the limitation of low sample size by having the surgeons repeating the rotation axis assessments on the same elbow specimens. This was done in a blinded fashion, so the surgeons were not aware that they were looking at the same elbow again.

Perfect fixator alignment remains a difficult procedure even for highly skilled surgeons due to variation between patients and due to natural variation of the elbow rotation axis during flexion–extension motion [12]. Some surgeons have switched to using a static fixator and no longer use a hinge, but many surgeons adhere to hinged external fixation because it enables early mobilization postoperatively [6]. In choosing the method to intraoperatively align fixator orientation, fluoroscopy is easy to use and requires only standard equipment. However, fluoroscopy seems insufficient to completely eliminate malalignment of external elbow fixators, given the rotation and translation errors described in this study. Preoperative CT may prove useful in the future to tailor intraoperative landmarks, similar to techniques used in knee arthroplasty [16]. For example, Sabo et al. [17] recently explored the value of the posterior humeral cortex on preoperative CT as a landmark to place the humeral component during elbow arthroplasty. As an alternative to fixator axis placement by the surgeon, hinged fixators can also be designed as self-centering devices. This recent development was shown to be effective in a study with seven patients, who all had correct alignment of the external fixator and had no complications [18]. Awaiting the introduction of such developments into common practice, we recommend adding the RD as anatomical landmark when using fluoroscopy for aligning hinged external fixators with the elbow rotation axis.
